# A User Centered Methodology for the Design of Smart Apparel for Older Users

**DOI:** 10.3390/s21082804

**Published:** 2021-04-16

**Authors:** Silvia Imbesi, Sofia Scataglini

**Affiliations:** 1 Department of Architecture, University of Ferrara, 44123 Ferrara, Italy; silvia.imbesi@unife.it; 2 Department of Product Development, Faculty of Design Science, University of Antwerp, 2000 Antwerp, Belgium

**Keywords:** user centered design, design methodology, usability, design for the elderly, ageing users, smart clothing, smart apparel, digital human modeling, biomonitoring, quality function deployment

## Abstract

Smart clothing plays a big role to foster innovation and to. boost health and well-being, improving the quality of the life of people, especially when addressed to niche users with particular needs related to their health. Designing smart apparel, in order to monitor physical and physiological functions in older users, is a crucial asset that user centered design is exploring, balancing needs expressed by the users with technological requirements related to the design process. In this paper, the authors describe a user centered methodology for the design of smart garments based on the evaluation of users’ acceptance of smart clothing. This comparison method can be considered as similar to a simplified version of the quality function deployment tool, and is used to evaluate the general response of each garment typology to different categories of requirements, determining the propensity of the older user to the utilization of the developed product. The suggested methodology aims at introducing in the design process a tool to evaluate and compare developed solutions, reducing complexity in design processes by providing a tool for the comparison of significant solutions, correlating quantitative and qualitative factors.

## 1. Introduction

Globally, populations are shifting their habits from “evolving societies” to “smart societies” that aim to unravel various social challenges by incorporating the innovations of the fourth technological revolution (e.g., Internet of Things (IoT), big data, artificial intelligence (AI), robot, and therefore the sharing economy) in every industrial sector and society. This kind of “evolution”, in the long run, impacts the way of managing social issues related to people’s well-being, offering innovative possibilities for the design of products and services which are created to make people’s lives better and more sustainable [1].

One of the main fields where the IoT and AI are demonstrating their great potential and usefulness in improving users’ quality of life, is the broad one of *health and wellness*, in proportion to various fields ranging from the field of sports to medical to styling etc. [2]. We are moving from the introduction of monitoring mobility on healthcare service (mHealth) through the utilization of wearable technology to embedded sensors in the ambient intelligent environment (a-health), finally to think about smart city-based healthcare [3].

In this ecosystem, smart clothing represents an intelligent device with an intimate and close relation with the body that is becoming an essential tool in older users. It plays a big role to foster innovation and to boost health and well-being, improving the quality of the life of people, especially when applied to users with particular needs related to their health status [4,5].

Intelligent garments can be used for measuring physical (body posture, gait impairments, fall detection, location (Global Positioning System, GPS) and physiological (cardiac activity (Electrocardiogram, ECG)), muscle activity (Electromyography (EMG), Respiration Rate (RR)) parameters in monitoring health status. These unobtrusive devices can be used for tracking and self-monitoring of people’s conditions in different environments, limiting the stress to be continuously monitored and observed [6]. In fact, smart clothing data can be connected to a tablet, a smartphone, or a smartwatch representing a virtual coach for elderly care and well-being. Smart clothing information can be shared through mobile health cloud systems with family, caregivers, and doctors, representing also a valid tool for remote tele-health and integrating alerting, improving quality of life at older ages.

Innovative technologies, as the ones related to smart clothing, are used to improve people’s quality of life, facilitating the actions of daily life in the most disparate areas, through the careful design of products, services, and processes [7]. The high possibility of personalization and the improved usability offered by technological innovations make it easier to try to fulfill specific needs expressed by users belonging to restricted categories of people as the one of ageing people. Particularly, the user centered design approach suggests to study and analyze specific kinds of users and define their peculiar requests and needs, interpreting them as design requirements that should be integrated with technical and technological ones [8]. For these reasons, user centered design is considered as a suitable approach for projects regarding the specific peculiarities of ageing users [9].

Older people are exposed to frailty syndrome that creates a vulnerable status that is associated with cognitive, motor, and psycho-social risk factors that cause falls, musculoskeletal injuries, neurosensory problems, hospitalization, and mortality [4,5].

The concept of ageing refers to the biological, psychological, and sociological transformations happening in every person in relation with the advance in chronological age. These changes, combined with environmental and cultural factors, impact on the person’s perceptual, cognitive, and psychomotor abilities [9], influencing the relationship with smart devices that often involve senses such as vision, hearing, and touch that are usually fundamental for an effective interaction [10].

While their abilities are decreasing, older users are called to face the use of smart new technologies, being open to an improvement of their digital skills; learning this kind of information, experimenting new ways of interaction with products and accept different habits, are critical points influenced by the level of satisfaction in use that is linked to the user capabilities.

Due to these considerations, designing for older people requires a big effort in analyzing their needs, also in the light of the individual health status, which is why it is necessary to extend the study of the category at several levels including different knowledge fields [11].

Particularly, design research related to smart garments should include the users’ voice at each stage of the design process in order to maintain a high degree of usability and wearability. These values are very important, especially for ageing people, because they ensure that the users take advantage of the product’s satisfying and fulfilling experience, which will help them to face, in a better way, a specific event or activity of their life [12]. The development of solutions able to improve elderly’s living conditions would empower the well-being of a big part of the current society, reducing significantly public expenditure in the fields of healthcare, medicine, and rehabilitation [13,14,15]. Even the World Health Organization (WHO) in recent years is highlighting how important it is to preserve and improve the elderly’s autonomy, recommending healthy and active ageing to postpone the necessity of personal and medical assistance, empowering motor and cognitive abilities, and reducing the pressure on the public health system [13].

Usually, one of the peculiarities of older users is the difficulty in the fruition of widely distributed products and services conceived for a user without impairments related to motor dysfunction or cognitive decline [16]. Health conditions impact significantly on users’ requirements to be satisfied from smart devices, consequently, health trends in an ageing population are conditioning the design and the technology addressed to the elderly user’s care, health, and well-being [17].

In this paper, the authors analyze several aspects of the user centered design process, dealing with the definition of requirements and constraints for the creation of smart clothing for older users, in order to improve the level of needs’ satisfaction they have expressed. The authors initially describe technologies related to the design of smart clothing. Then, they propose a methodology for the comparison between smart garments in order to evaluate advantages and disadvantages, to focus on which aspects can be effective for the acceptance of smart apparel by ageing users with specific needs, related to impairments affecting the physical dimension. Developing tools for the evaluation of the impact of factors as functionality, maintainability, connectivity, usability, wearability, accessibility, aesthetics, and emotional, allows to analyze how “design effective devices” can really increase users’ quality of life.

## 2. Methods

Involving users in the whole design process of smart garments, as recommended by UCD, lets the design team focus on the issues related to the physical (and cognitive) interactions between the user and the object: smart clothing for older users requires particular properties of wearability and usability to be accepted and used by the older person. Thus, it is necessary to design clothes that are addressed to the orders’ needs, looking at their physical, sensory, and cognitive abilities and impairments [18,19].

In order to better understand which are the values considered for the definition of constraints and critical aspects, it is important to express several definitions starting from usability, intended as the capacity of a product or a service or a system to provide to its users the conditions to perform tasks safely, effectively, and efficiently while enjoying the experience [20]. In relation to usability, there is ease of use which describes how easily a user can use a product following the specific metrics of the project [21]; accessibility as “ability to access” and benefit from the product, service, or system, focusing on enabling access to people with special needs or impairments [22]; user-friendliness as the attribute of something which is easy to use, clean, intuitive and reliable [23]; and finally, sustainability as ecological and social equity and consciousness [24].

Together with the values described for user centered design, there are other design aspects mostly related to technology and manufacturing/production; among these issues are significantly relevant the ones regarding fabrics that should be hypoallergenic, washable, stretchable, thermoregulatory breathable without affecting mobility enhancing comfort and well-being of the user. Fabrics should be assembled in order to obtain garments able to maintain adherence to the body (e.g., at chest level at 10th rib) for maintaining the close interaction of the cloth to the skin in the case we are using textile conductive electrodes (textrodes) for monitoring ECG signals. An improper adherence of the textrodes to the skin can cause signal artefact.

Considering technological issues, we can highlight the obvious safety of the device; reliability as the ability of the product to perform a specified function within a given environment for an expected lifecycle [25]; effectiveness described as the capability to produce the desired output [26]; efficiency as the optimization of resources as materials and components, to provide the maximum quality at minimum cost; and finally, durability as the capacity of resisting time.

In the design process, the combination between technological and design issues is a fundamental asset for developing an effective smart clothing device. Moreover, each issue is complementary to the other, influencing it and exerting a strengthening or weakening influence depending on the efficiency of the match between requirements and developed solutions.

Next to the user centered design and technological aspects, there are even other tangible characteristics related to the perception of formal aspects as shape, color, tactile consistency, etc. and influences the user’s feedback on the device because of its impact on empathy and emotions. Moreover, personalization according to the aesthetic and formal preferences of the person allows identification with the design product which greatly influences acceptance and propensity to use [27].

The balance and the combination between those three groups is strategic in the design of smart garments for the elderly. This category of users could gain a lot of advantages in the involvement within the design process, considering that currently, common standards for the design of smart devices and smart garments are not based on elderly’s bodies or necessities. For these reasons, constraints or critical points in the entire design process should be managed by simultaneous management of the three groups of design aspects and considering the relationship between them and the final user [28].

**Figure 1:**
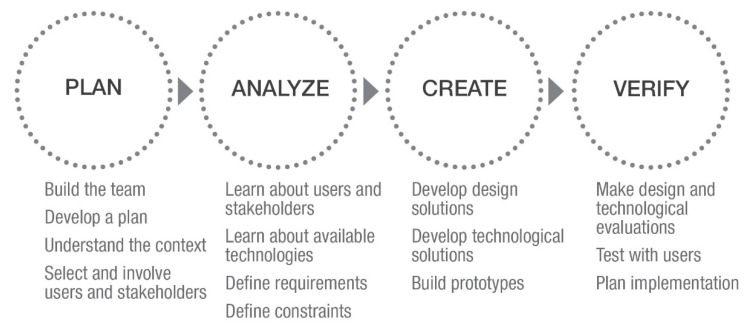
Scheme representing the design process proper of the user centered approach, as intended by usability.gov [29].

Considering the design development of smart apparel for older people, it is suggested to apply the typical user centered design process that is composed of four main design stages: planning, analyzing, creating, and verifying (Figure 1).

In the planning stage, the multidisciplinary team that is composed of experts in this domain is in charge of planning and identifying the context for primary and secondary users and stakeholders. Next is the analyzing step where an in-depth analysis of users’ requirements and technological constraints is carried out, together with an evaluation of competitors (benchmarking). During the creating stage, the team develops several design solutions thanks to the application of available technologies and combines them for the creation of real prototypes for testing different aspects. The last part of the design process is verifying, where it is possible to make evaluations on design and technology thanks to the testing phase with users and to the application of several technological tools [30].

As previously described, the user centered garment workflow involves elderly users and their needs in the entire pathway, but especially in the analyzing and testing phases. During the process, collected needs are translated into requirements and combined with constraints for the definition of a design project plan, leading to the definition of the design project and to the realization of rough and advanced prototypes for different typologies of evaluations.

Evaluations and tests involved in the last part of the design process are the most important feedback for the assessment of requirements satisfaction, for the verification of correct application of constraints, and for the resolution of critical issues emerged during the realization and testing of prototypes.

Nowadays, there are design and digital tools available for the verification of the design project that can be used to optimize all the issues related to the realization of smart clothes such as usability, wearability, accessibility, etc.

In this paper, authors are proposing a methodology for the evaluations of different quantitative and qualitative aspects of a smart clothing project. This approach can be used in the iterative design process from a multidisciplinary team to manage, compare, and hierarchize all the different feedback collected during the testing phase from involved ageing users. Moreover, this methodology allows the definition of points of strength and weakness of the evaluated garment from the users’ point of view.

### 2.1. Clothing Design from Traditional to Virtual

**Figure 2:**
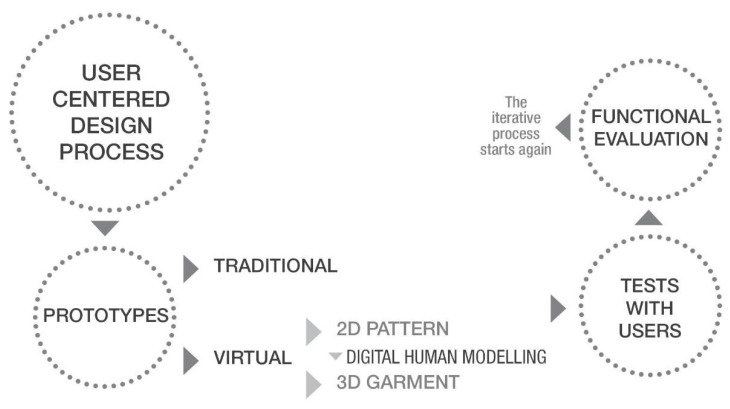
Digital human modeling (DHM) contribution in passing from 2D pattern to 3D garment.

Clothing design includes three main steps: style design, construction design, and process design. Firstly, the fashion design starts with initial illustrations, then the patternmaker makes the garment as flatting drawing and grading in a traditional way that sometimes reveals to be time-consuming and inefficient. Virtual prototyping is able to solve that allowing the designer to create the first prototype from the 2D pattern then creating the 3D shell in the virtual environment using digital human models (Figure 2).

Digital human modeling (DHM) refers to digital tools for virtual prototyping and simulation of aesthetics, fitting, and virtual try-on [31,32]. DHM can serve either as a virtual try-on for the user or serve as a fashion designer for simulating and finalizing the product development during the refining phase. Those characteristics make DHM an effective tool for the development of user centered projects, especially if addressed to older users needing special attention for issues regarding their physical impairments restricting movements. Older users often look at virtual prototyping as a problematic issue because they are not used to interacting with innovative technologies and sometimes have difficulties in understanding properly how many advantages they can have from the possibilities of personalization provided by smart devices.

DHM can be a precious tool to solve elderly’s problems related to fitting aspects, using a virtual try-on or customization including personalization. Moreover, benefits of using virtual garments prototyping are also related to assessing zero-waste design (sustainability), eliminating the sewing process, representing a quick response for garment design.

The virtual design process starts with the identification of the body shape. This can be done using anthropometric surveys that contain scanned subjects of a specific country using a specific body scanner [33] or scanning the subject directly in real-time. As an alternative, it is possible to use a reconstruction process from images using a dedicated application (APP) in a smartphone or tablet [34].

Starting from the shape, it is possible to realize a statistical shape modeling (SSM) mapping the body shape variability of anthropometric databases of 3D body scan [35,36]. Using the SSM and a bio vision file (bvh) captured using a mocap system in older users, it is possible to replicate a real motion activity in SSM, creating a kinematic model.

Nowadays, there are many software applications available (e.g., Clo 3D [37] Optitex [38], Gerber [39], YUKA [40], Lectra [41]) for creating virtual clothing 3D simulations but they are costly and sometimes difficult to use. Open Source software such as Blender can be an option for this realization with some limitations but also many benefits. It is possible to replicate a human activity (e.g., an older user who is wearing a garment) into a virtual environment constituting a kinematic model. Scataglini et al. (2018, 2019) [31,32,35], described the workflow for realizing a kinematic model and the dressing up starting from SSM.

An example of the steps that can be used for this realization is explained below. In this case, a body shape was realized using the open-source software Makehuman [42]. The authors used this software for showing the steps necessary for creating the kinematic model. Of course, Makehuman is a software that cannot be used for real clothing customization.

**Figure 3:**
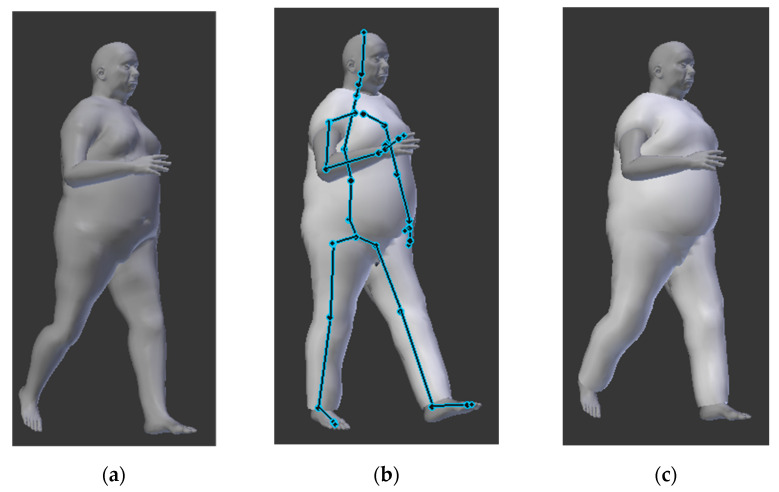
Kinematic model in Blender ((**a**) statistical body shape model, (**b**) parenting of the mesh with the armature, (**c**) rigging of the statistical shape model with the cloth and the bvh file).

Once the body shape is realized, it is possible to import this into Blender (Figure 3) as Obj. file, together with the 2D pattern, creating the 3D shell.

Successively, it is possible to acquire a real activity or specific movement of the user (e.g., the walking of the older user while he is wearing a smart shirt or trousers) using a wearable inertial mocap system. This can be imported as a BVH file (bio vision file) into Blender. Afterward, we use UV mapping with the body mesh and the rigging of the shirt or clothes (Figure 3) with the kinematic model [35].

Feedback derived from this design phase is fundamental for the realization and for the prototyping phases because they can substitute expensive and lengthy real prototyping realizations, whose fitting can be stressful and painful for users involved in the experimentation. DHM allows designers to save a significant amount of resources, giving a very precise and accurate level of simulation of the body of the person and guaranteeing an effective verification of ergonomic aspects involved in the project of the specific smart cloth [35].

### 2.2. Technological Issues

Smart clothing is the result of the interaction and integration of two borders: the electronic and the textile one, becoming an indispensable electronic textile (e-textile) for real non-intrusive monitoring.

**Figure 4:**
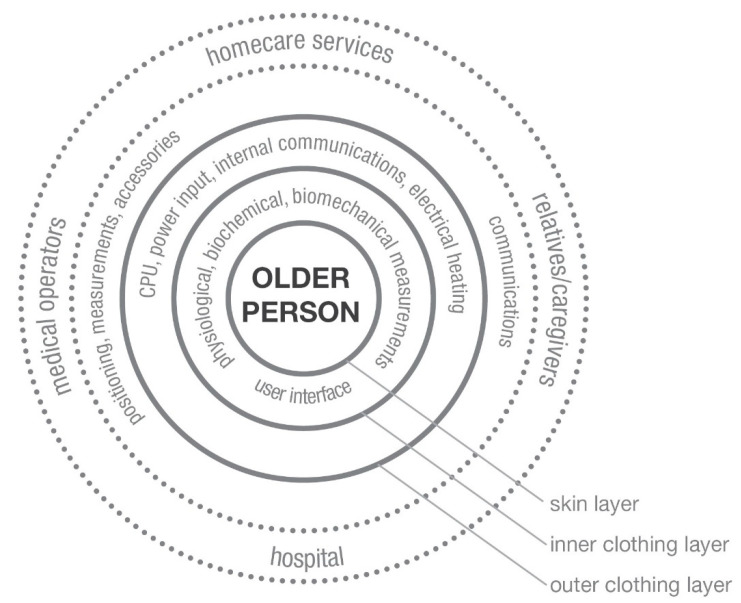
Evolution of the concept model of smart clothing for older users [43].

The interaction between layer and border can be active (sensoric, adaptive, self-healing) or passive (as a barrier against cold, rain, and wind). In fact, according to the conceptual model of Ranten and Hännikäinen [38], there is an inner layer that is close to human skin (Figure 4). This layer can be used for physiological, biomechanical, biochemical monitoring. Then there is an outer layer that can serve as a barrier to external agents (e.g., atmosphere, etc.). Communication can be done internally, spatially, and externally using interfaces as snaps, connectors, fixed support, and switches.

If the communication passes through the layers, it is called wearable body area network (WBAN). When the communication from the layers is acquired by aggregators such as smartphones, tablets, a server or a cloud, it is defined as personal area network (PAN).

This communication is in a short range (10 to 100 m) using an example ZigBee and Bluetooth. Alternatively, the connection can be also to a local area network (LAN) using Wi-Fi to a remote server as the patient’s personal home server (PPHS) or cloud.

Between these systems and subsystems that interact with the human and their environment, smart apparel constitutes an essential tool for supporting the older user’s health and care services from home to clinical settings, involving general practitioner, relatives, and clinical care teams in the hospitals.

**Figure 5:**
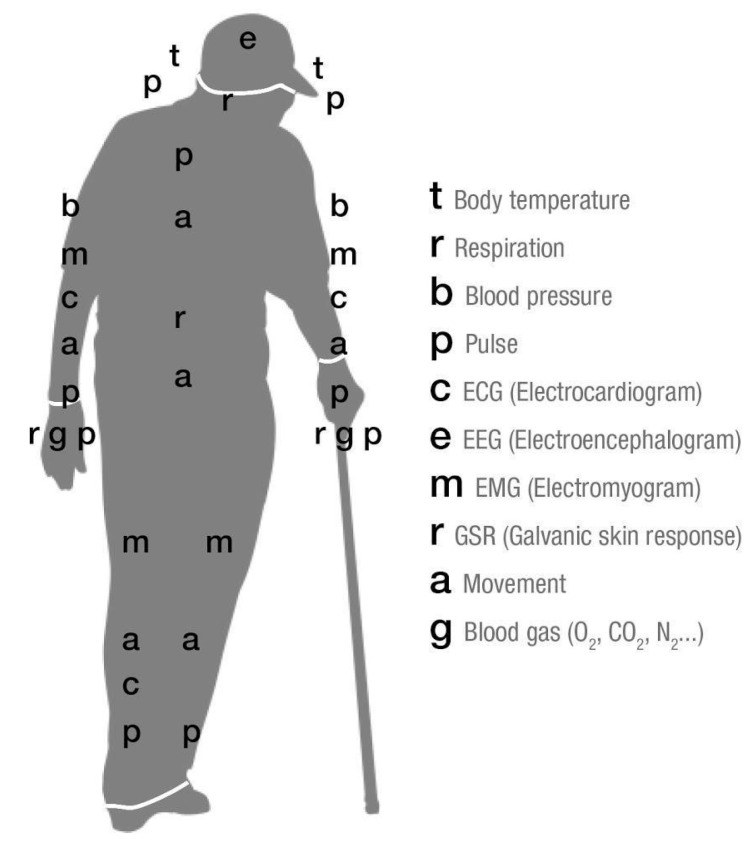
Biomedical signals that can be measured in the human body [44].

The sensing subsystem can contain different sensors for measuring biomedical signs of the wearer (Figure 5). The most suitable sensor location should consider stable positions that do not create artefact of movement or interactions with aids.

In terms of physical signals as gesture, motion, posture, and activity, the most used sensors are accelerometers or wearable inertial measurements units (IMU) that can be connected or integrated into the clothing by snaps, straps, or fabric pockets. IMU integrates an accelerometer, magnetometer, and gyroscope in a unit able to quantify lower joint kinematics if an example is integrated in trousers or upper body kinematics if they are integrated into a smart shirt.

Alternatively, nowadays, it is possible to use one single IMU sensor or accelerometer for determining gait and integrate them in a smart belt (L5-S1, lumbosacral joint), shirt, or bra (at chest level) [45,46]. In the case of older users, the smart bra in terms of autonomy is considered not a good way to monitor them as it is not simple to wear. The shirt and the belt can additionally be probably more accepted by the user in terms of autonomy. The belt respects the shirt is simple to wear but is affected by physical variability in body shape associated with an excess of fat around the belly.

Smart clothing can be used for monitoring cardiac activity (ECG), substituting silver/silver chloride (Ag/AgCl) electrodes by using textile yarn, that as yarn with electro-conductive fibers (polymeric or carbon coated threads) or metal yarns (that contains conductive fibers as copper, stainless steel, silver with synthetic or natural fibers). Textile sensors or textrods, with respect to electrode, preset the capability of monitoring the subjects without using conductive gel that can result uncomfortable on a sensitive skin especially when we are using it for long term monitoring [45,46,47].

Textile strain gauge sensors can be applied at the chest and to the thorax for measuring respiratory activity.

For location and tracking, it is possible to use a GPS wearable textile antenna integrated into a cloth such as a hat to form a GPS tracker location. This is important for monitoring dementia or Alzheimer in older adults.

While for measuring temperature, it is possible to use microsensors as laden textile embedded to the cloth as socks for measuring and monitoring the temperature in pathology such as the diabetic foot; or smart polymeric optical fibers (POF) for measuring not only pressure but also frictions that can be used in older adults.

For enhancing comfort, we can use the smart coatings as phase change materials (PCM) that can respond to external stimuli passing from a state to another state as solid to liquid producing a thermoregulatory effect. The property of this material as the Outlast technology developed by NASA is to absorb or release heat maintaining a thermo-physiological comfort.

### 2.3. Functional Evaluation

Functional evaluation is extremely important in the user centered process for designing smart garments constituting a retro feed-back in the design process [17].

This retro feedback is composed of the evaluation of physical and physiological monitoring of the potential user during different tasks, wearing smart apparel in a real and virtual environment (using DHM). This constitutes valid feedback in the iterative design process involving the user also.

Once the first prototype has been made, the project team can test it with specific end-users, representing a sample of the target users, to evaluate and get some insights to implement it. At this stage, it is possible to ask the old user to answer a tailor-made questionnaire that can further determine the critical points that can help figure out user acceptance. Usually, this kind of questionnaire is composed by a part of multiple-choice questions on functional aspects and the performed activity, and a part with open questions where the user can express his impressions and feeling about the testing experience.

The project’s fulfilment of user’s requirements can be checked by verifying the response of the final prototype to the users’ needs which were defined as crucial at the beginning of the design process. This approach can be even applied in an innovative way when we have to design a smart device responding to specific needs, but not necessarily belonging to a specific product category.

In this situation, it is possible to compare how existing devices respond to those given needs, in order to formulate evaluations on possible innovative solutions that keep the most effective solutions from different products already prototyped and tested.

There are really many ways of measuring the compliance of a smart project to the given requirements, the authors chose the one proposed by Tsai-Hsuan Tsai et al. [48], as significant for the evaluation of the elderly’s needs satisfaction. The cited protocol proposes the evaluation of important aspects related to the device, by elaborating a group of questions for each aspect and asking a user to answer by marking. The authors modified the original table, making it suitable for the evaluation of smart clothing for older users, by adding and modifying some questions in order to highlight significant aspects that can be relevant in the functional evaluation.

Using this interview for a specific user regarding a specific smart garment, makes it possible to figure out older people’s needs regarding different aspects of the relationship between the person and the smart cloth such as aesthetics anxiety, comfort, technology anxiety, perceived ubiquity, resistance, perceived usefulness, perceived ease of use, attitude, and behavioral intention.

## 3. Results

Given the previous considerations, authors decided to evaluate some example cases of different kinds of smart garments, in order to observe advantages and disadvantages of each typology for the fulfilment of users’ requirements. Moreover, the following table allows the comparison between different devices on several common aspects regarding their perception from the elder’s point of view.

Starting from this, it is possible to make considerations and suggest design strategies to improve the effectiveness of smart solutions, thanks to their high ability in satisfying requirements expressed by the users and in overcoming critical issues given by constraints.

### 3.1. Smart Garment as Shirt, Bra, Socks, Hat for Elderly

Concerning the comparison between different typologies of smart garments, the authors selected five case studies from a systematic review of scientific literature regarding innovative smart apparel not specifically conceived for older users but with interesting features or functions for them. The systematic review was pursued using the keywords “smart clothing” and “elderly” searching in three databases as IEEE, WOS, and Pubmed according to the PRISMA guidelines. From the first screening after the doubles were removed, a total of 118 articles were identified. After screening titles, abstracts, and full papers, 33 papers were identified according to the selection criteria based on population(elderly), intervention (only studies that contain smart clothing), outcome (studies with the analysis of health and well-being in elderly), design (full text excluding systematic review), and language (only article written in English). From this final identification, five devices were identified and included in this review.

All the chosen devices were designed for the ambulatory environment and should be used for the older person by an operator or caregiver, in order to monitor some characteristics or activities of the user. All smart garments do not require a great effort to be worn and are not conceived to be worn for a long amount of time. Finally, the selected smart clothes responding to these specifications are a shirt, a bra, socks, gloves, and a hat.

The first selected device is a smart vest developed by Lin et al. (2018). The smart shirt is connected to the cloud and to a mobile device of a doctor. Four textrodes are embedded into the garment for ECG monitoring. The device acts as a device for surveillance, like when the ECG signal is above 140 Hz or lower than 50 Hz, an alert signal is triggered to the medical unit; or it can be used to alert when the subject is falling or emerging using a snaps when the elderly needs it [49].

The second device is composed of a smart bra, a smartphone, and a smart watch. The smartphone, worn around the neck, is able to monitor acceleration and location. In addition, the system is able to identify the person in a group also showing the interaction to the smartwatch that vibrates to display the picture of the person. The bra also presents two textrodes at the chest level for heart rate detection [50].

The third device is a smart sock for monitoring temperature in diabetic neuropathy. Thin flexible fiber optic sensors are woven into the socks for measuring temperature and pressure under respectively, big toe, first metatarsal head (MTH), fifth MTH, midfoot, and hind foot [51].

The fourth, is a smart glove, with transcutaneous electrical nerve stimulator (TENS) for the elderly’s hypertensive treatment. The device presents an inner with e-textile for stimulating meridian points on the palm, an arm band for ubiquitous healthcare, and TENS [52].

The fifth, is a smart system for the indoor tracking of the gait of the person, using a smart hat and a smart shoe for monitoring the movements as an AAL device [53].

### 3.2. Comparison between Smart Clothing Typologies and Related User Needs

The five smart garments previously described have been compared in a matrix with the needs extrapolated by the table of user’s requirements for functional evaluations [48] (Table 1).

This comparison method can be considered as similar to a simplified version of the quality function deployment (QFD) tool [54], which is used to evaluate the general response of each typology of cloth to the different categories of requirements, determining the propensity of the older user to the utilization of the developed product.

Many alternative versions of the QFD have been elaborated in recent years in order to adapt this user centered tool to new objectives, as the use in multidisciplinary teams or the design and development of services and systems related to products [54]. In this particular case, the aim was a comparison between different products and not between characteristics, making it similar to a benchmarking QFD [55].

In this context, the authors propose an evaluation matrix that integrates the needs elaborated previously by Imbesi et al. [12,56] with the acceptance model proposed by Tsai-Hsuan Tsai [49] to evaluate the smart clothing acceptance.

The five garments identified in the scientific literature were evaluated, attributing a score from 1 to 3, expressing how much that object is able to satisfy the specific need (1 low satisfaction, 2 neutral satisfaction, 3 high satisfaction). The scale 1–3 was chosen in order to have a balanced distribution of marks, avoiding some ambiguities deriving from difficulties in assigning marks from a larger scale. The activity of attributing a score to each need was done by authors considering single devices’ peculiarities reported in related publications.

For every typology of smart apparel, it was analyzed how it could respond to the need expressed by the user. For needs regarding practical aspects, it was easy to understand the level of user’s satisfaction by considering the description of the single device (e.g., “I don’t want it to be too tight for my body shape”: smart shirt and smart bra are conceived with adjustable closure systems that allow different sizes; on the other hand, socks, glove, and hat at the moment have a unique size fitting a medium user). For needs concerning feelings and emotional aspects, the mark was given considering scientific literature and previously cited research experiences [12,56,57] (e.g., “I want it to improve my life quality”: we can hypothesize that all devices will because they are all conceived to monitor health parameters in an efficient and non-invasive way, in order to improve the person’s assistance).

## 4. Discussion

After marking each intersection gap of the matrix, scores belonging to the same group of needs have been added and results are reported with a percentage that considers the achieved degree of satisfaction (the real marks), compared to the potential one (the total amount of marks). These percentages are calculated and compared in order to observe how different typologies of clothing are able to satisfy different categories of values (Table 2).

This matrix makes it possible to hypothesize considerations on the reasons why a typology of smart clothing is more effective than another in the satisfaction of a specific group of users’ needs.

This methodology can be applied to the testing phase for garment design, constituting a valid procedure for analyzing ageing users’ acceptance. Each separate variable analyzed is then associated to a value expressed in percentage, identifying the level of acceptance of the smart apparel for older users.

The smart shirt gains a high score in the satisfaction of the majority of categories of needs. It is possible to suppose that one of the main advantages it offers is related to wearability. The possibility of adjusting the size and physicality of the reference user, makes this cloth versatile and adaptable as it was personalized. As with most smart devices, one of the weakest points is anxiety to use technology and perceived ease of use, because of difficulties of older users in feeling comfortable with products with a high level of technological innovation.

The smart bra is perceived as a useful device because of its functions, but at the same time, it feeds the user’s resistance to accept an innovation that greatly influences perception in everyday life. Its utility is revealed in the high score for behavioral intention, because the user is allowed to have significant feedback, letting him perceive how the smart cloth is able to influence positively the action related to daily activities.

The smart socks can satisfy needs related to perceived ubiquity as most devices developed to monitor health conditions, postural positions, and movement. Comfort and positive behavioral intention are strong points thanks to the non-invasive and ecological approach. However, the device is not perceived as easy to use because of the lack of feedback it can give to the user.

The smart glove feeds technology anxiety and is not perceived to have ease of use, primarily because of the position: the majority of devices used on hands makes the user perceive it as something reducing mobility and grip, even if in scores related to comfort the user admits that the glove is not intrusive at all. On the other hand, perceived usefulness and behavioral intention are good because the user can understand the garment’s utility for monitoring the health condition.

The smart hat has good marks in perceived ubiquity because of its monitoring functions, and in behavioral intention thanks to the readability of functions for the user. Aspects where scores are low are technology and aesthetics anxiety, the reason is that the device, being on the head, does not seem controllable by the user, and the possibility for everybody to see it makes the user feel observed and judged as an impaired person by other people seeing him using the device.

Considering resulting percentages, it is possible to identify both positive aspects to be maintained and developed in the design of new smart garment typologies, and not satisfying issues needing a design implementation in order to gain a better level of ageing users’ acceptance.

The characterization expressed by these descriptions, is used to have a wide vision of which are the most significant peculiarities to keep in consideration for every smart cloth when we address it to an elder user. These considerations can be used to choose a typology of smart garment instead of another, based on how it is focused on the specific needs expressed by the older user. The confrontation of different garments can be done in a quantitative and not only qualitative way, considering several aspects that are difficult to compare because of their diversity.

Moreover, once the typology is chosen, it is possible to understand which are the critical aspects that are not satisfying properly the established requirements. The correct identification of critical aspects and the comprehension of functional and emotional factors that induce criticalities, are correct strategies for the empowerment of the design of smart clothing.

## 5. Conclusions

Smart clothing is an innovative tool for monitoring health, care, and well-being during daily life activities; these are the reasons why it can give a great contribution in improving elderly’s quality of life, by empowering their autonomy and independence.

User centered design has a great role in the design process of smart clothing for its ability to understand the user’s needs, elaborate a strategy to satisfy them, and guide the elder person’s perception to a positive attitude.

The modalities in which the user is involved, and the interpretation given in understanding his/her feedback, condition and modify the entire design process. The project phases are usually carried out by a multidisciplinary team involving professionals with different backgrounds that interact with selected users, with the aim of collecting and interpreting their needs and feedback on the device.

The combination between different fields of knowledge implies an arrangement of qualitative and quantitative factors, in order to evaluate processes, design solutions, typologies, technologies, etc. The scores expressed by members of the multidisciplinary team in the matrix, are a balanced vision of the different fields of knowledge involved in the design process.

This user centered methodology has the aim of reducing complexity in design processes by providing a tool for the comparison of significant solutions. The possibility of combination and correlation of quantitative and qualitative factors is the first step towards the overcoming of complex issues related to the project.

The shown method is actually used for the evaluation of distinct typologies of smart garments, but it can be used even to compare different characteristics of a single product, or in order to choose a possible design solution instead of another, allowing several evaluations depending on the scope of the design project.

The use of this methodology can be experimented even in other design fields where the way of combination of quantitative and qualitative factors influences the whole design process, due to the resolution of the project’s complexity.

## Figures and Tables

**Table 1:** Acceptance evaluation matrix for smart apparel.

		Smart Shirt	Smart Bra	Smart Socks	Smart Glove	Smart Hat
Aesthetics Anxiety	I want its color to be good for my age.	3	3	2	2	2
	I don’t want it to be too tight for my body shape.	3	2	3	2	3
	I don’t want to be afraid to wear it with other clothes.	2	1	2	2	3
	I want to put and remove it without help.	3	2	1	2	1
	I want it to be in a material I feel comfortable with.	3	3	2	1	2
Comfort	I want it to be my size.	3	3	2	2	2
	I don’t want it to reduce my mobility.	3	3	3	2	3
	I don’t want to get too cold or too want wearing it.	2	2	2	2	2
	I want it to be breathable.	3	3	3	2	3
	I want the material to be soft.	3	3	3	3	3
Technology anxiety	I don’t want to be apprehensive about using the smart cloth.	2	2	2	1	2
	I don’t want the fear of making mistakes that I cannot correct.	1	2	2	1	1
	I don’t want the equipment to suddenly stop functioning.	2	2	2	2	2
	I don’t want other people to see me wearing smart clothes.	3	1	3	1	1
	I don’t want it to interfere with other aids or devices (e.g., pacemaker, hearing aids,...).	2	2	2	2	2
Perceived Ubiquity	I want it to provide health care information anytime anywhere.	3	1	3	3	2
	I want it to provide me anytime and anywhere communication and connectivity.	3	2	2	2	2
	I want to use it for healthcare purposes.	3	3	3	3	3
Resistance	I don’t want it to change the way I deal with related problems.	3	2	2	2	3
	I don’t want it to change the way I keep myself healthy.	3	3	3	3	3
	I don’t want it to change the way I interact with other people.	3	2	3	2	2
	I don’t want it to change the way I currently live.	2	1	2	2	1
Perceived Usefulness	I want it to improve my life quality.	3	3	3	3	3
	I want it to make my life more convenient.	3	3	3	3	3
	I want it to make me more effective in my life.	2	3	2	3	3
	I want it to be useful in my life.	3	3	3	3	3
	I want it to alert my family or caregiver when I am alone.	3	2	1	1	2
Perceived ease of use	I want it to be clear and understandable.	3	3	2	2	2
	I don’t want it to require a lot of mental effort.	2	1	2	2	2
	I want it to be easy to use.	1	2	2	2	1
Attitude	I want to think that using it is a good idea.	3	3	3	3	3
	I want to think that it is beneficial to me.	3	3	3	3	3
	I want to have a positive perception of using it.	2	2	2	3	1
	I want it to make me feel safe.	2	3	1	2	3
	I want to use it for health but also for gamification.	1	3	1	1	1
Behavioral Intention	I want to use it in the future.	3	3	3	3	3
	I want to use it always in my daily life.	2	3	2	3	1
	I want to use it every time I am sleeping.	2	1	3	3	1
Maintainability anxiety	I want to be able to wash it effectively without issues.	2	2	1	1	1
	I want it to last as long as I need it.	2	2	2	2	2
	I want to be able to replace elements (battery, sensors…) without breaking it.	2	2	1	1	1
	I want its disposal to be easy and sustainable.	2	2	1	1	1

**Table 2:** Table of results of the comparison between smart garments.

	Smart Shirt	Smart Bra	Smart Socks	Smart Glove	Smart Hat
aesthetics anxiety	93%	73%	67%	73%	60%
comfort	100%	67%	89%	89%	78%
technology anxiety	67%	60%	60%	46%	53%
perceived ubiquity	100%	67%	89%	89%	89%
resistance	91%	67%	83%	75%	75%
perceived usefulness	93%	93%	80%	87%	93%
perceived ease of use	67%	67%	67%	67%	56%
attitude	73%	93%	67%	80%	73%
behavioral intention	78%	78%	89%	100%	56%
maintainability anxiety	67%	67%	42%	42%	42%

## Data Availability

Not applicable.
